# Correlations in Magnetic Sub‐Domains as an Unconventional Phase Diagram for van der Waals Ferromagnets

**DOI:** 10.1002/advs.202500562

**Published:** 2025-04-11

**Authors:** Sergey Y. Grebenchuk, Magdalena Grzeszczyk, Zhaolong Chen, Makars Šiškins, Vladislav Borisov, Manuel Pereiro, Mikhail I. Katsnelson, Olle Eriksson, Kostya S. Novoselov, Maciej Koperski

**Affiliations:** ^1^ Institute for Functional Intelligent Materials National University of Singapore Singapore 117544 Singapore; ^2^ Department of Materials Science and Engineering National University of Singapore Singapore 117575 Singapore; ^3^ School of Advanced Materials Peking University Shenzhen Graduate School Shenzhen 518055 China; ^4^ Department of Physics and Astronomy Uppsala University Box 516 Uppsala SE‐75120 Sweden; ^5^ Institute for Molecules and Materials Radboud University Heyendaalseweg 135 Nijmegen NL‐6525 AJ Netherlands; ^6^ Wallenberg Initiative Materials Science for Sustainability, Department of Physics and Astronomy Uppsala University Uppsala 75121 Sweden; ^7^ School of Science and Technology Örebro University Örebro SE‐701 82 Sweden

**Keywords:** 2D magnets, correlations, ferromagnetism, phase diagram

## Abstract

Traditional magnetic phase diagram represents a transition between the ferromagnetic and paramagnetic states of a material under the influence of varied temperature, magnetic field, and pressure. So far, the ferromagnetic phase has been considered predominantly as a single type of magnetization texture extending macroscopically in the bulk of a crystal, existing as a ground state determined by the interactions between localized magnetic moments arranged in a lattice. Here, it is demonstrated that an unconventional magnetic order composed of vertically correlated planar magnetic sub‐domains occurs intrinsically in mechanically exfoliated layers of van der Waals ferromagnet CrBr_3_. Based on the visualization of the magnetic textures through magnetic force microscopy in conjunction with the ab initio calculations of the crystal structure in the magnetic phase and micromagnetic simulations, the origin of the magnetic sub‐domains is attributed to stacking faults isolating a van der Waals ferromagnetic well from the bulk film due to modifications in the interlayer exchange coupling. This enables to create a phase diagram describing the magnetic states unique to van der Waals ferromagnets in terms of the degree of correlation between the magnetic sub‐domains, dependent on the exchange coupling constants and tuneable by magnetic field and temperature.

## Graphical abstract

An unconventional magnetic order is observed in exfoliated CrBr_3_ layers, characterized by vertically correlated planar magnetic sub‐domains. Magnetic force microscopy, ab initio calculations, and micromagnetic simulations indicate that these sub‐domains arise from stacking faults modifying interlayer exchange coupling. A phase diagram is developed to describe unique magnetic states in van der Waals ferromagnets, tunable by magnetic field and temperature.

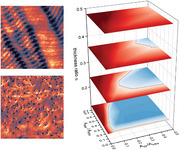

## 1 Introduction

Ferromagnetism belongs to a class of quantum phenomena that became prevalent in modern technology, where magnetic materials found applications in memory devices, computation, and medicine. The fundamental concept of ferromagnetism relies on the macroscopic arrangement of localized magnetic moments forming a lattice, most commonly originating from transition metal atoms/ions exhibiting unpaired electrons occupying *d*‐type atomic orbitals. The magnetic order exists as a ground state of an interacting system of magnetic moments via short‐range (e.g., exchange coupling), and long‐range (e.g., dipolar coupling) interactions under the condition of suppressed thermal excitations of spin waves below a critical temperature. As such, the formation and textures of the magnetic order strongly depend on the dimensionality of the lattice of magnetic moments and spin anisotropy.^[^ ^1^ ^]^ Recent developments in van der Waals materials exhibiting ferromagnetism down to single‐molecule‐thin films created a need for a universal description of magnetic ordering, that takes into account a broader variety of magnetization textures that can, in principle, occur in two‐dimensional systems.^[^ ^1^, ^2^, ^3^, ^4^, ^5^ ^]^

In this study, we demonstrated via magnetic force microscopy (MFM) that an unconventional magnetic order existed intrinsically in insulating van der Waals ferromagnet CrBr_3_ in the form of vertically correlated magnetization sub‐domains. We identified the origin of the multi‐domain structure as a modification of the interlayer exchange coupling at stacking faults between two‐dimensional layers. We quantified the degree of correlations between the two sub‐orders with a similarity index function, providing a macroscopic parameter describing the magnetic state of the sample. By confronting the MFM images with the results of micromagnetic simulations, we constructed a phase diagram where the similarity index indicated correlated and anti‐correlated phases unique to van der Waals magnetic systems. Consequently, we identified the sufficient conditions for the occurrence of correlated sub‐domains: 1) significant anisotropy between the intraplane and interplane exchange coupling constants, 2) quasi‐degenerate structural configurations in the magnetic phase enabling stacking faults that lead to intrinsic isolation of thin magnetic films from the bulk crystals, and 3) modifications of the interlayer coupling constant from ferromagnetic to antiferromagnetic at the stacking fault. The degree of the correlations expressed as the similarity index was dependent on the external magnetic field and temperature. Consequently, the emergent phase diagram describing the correlated phases arises as an essential tool for understanding the complex behavior of the ferromagnetic van der Waals films, heterostructures, and devices.

We envisage that the concept of magnetic phases comprising correlated sub‐domains can be universally applied across the family of 2D magnets that fulfill the specific conditions related to the structural and magnetic energetics. Our results point toward the natural formation of ferromagnetic wells in van der Waals systems, where a thin film decoupled from bulk exhibits vastly different magnetization textures. The existence of such structures opens questions regarding the confinement of elementary particles or topological textures in ferromagnetic wells, such as excitons coupled to magnetization, magnons, magnetic polarons, or skyrmions.

## 2 Results

### 2.1 Evidence of Coexisting Magnetic Textures in Exfoliated CrBr_3_ Films

**Figure 1:**
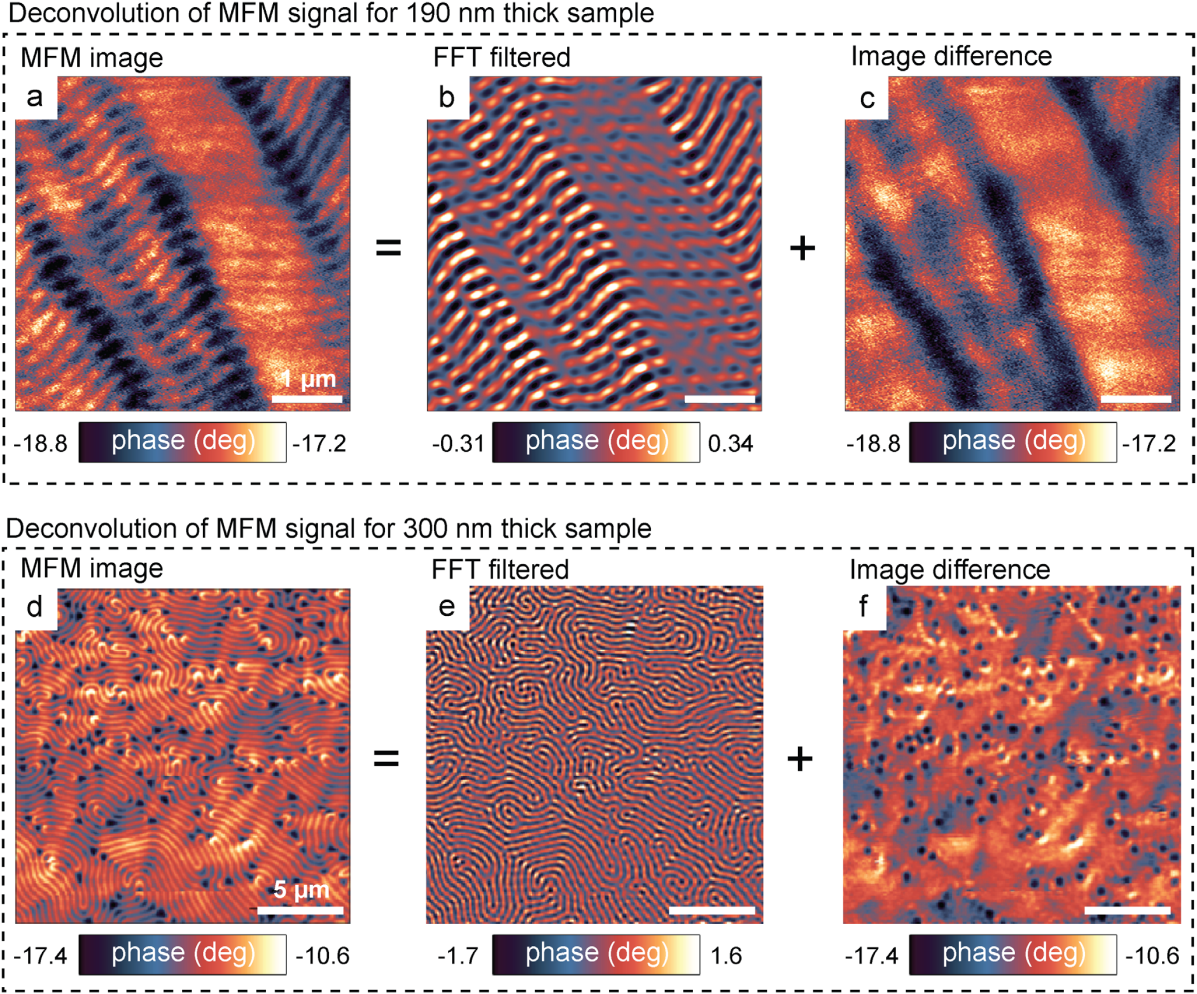
Coexistence of several domain sub‐systems. a,d) MFM images obtained from CrBr_3_ crystals with thicknesses of 190 nm (a) and 300 nm (d), demonstrating complex patterns consisting of multiple systems of domains. Image (a) was deconvoluted into figures (b) and (c), and (d) into (e) and (f) using FFT filtering to highlight several overlaying domain patterns. The similarity index for domain sub‐groups (b) and (c) is ξ = 0.26, and for (e) and (f) is ξ = 0.13. The sample‐tip distance was 50 nm for (a) and 100 nm for (d).

We investigated the magnetic properties of mechanically exfoliated CrBr_3_ layers deposited on Si/SiO_2_ substrates with the film thickness varied across over two orders of magnitude from a monolayer limit (around 1 nm thickness) to 600 nm bulk systems. We visualized the emergent magnetization textures via MFM imaging. This technique detects local changes in the magnetic stray field through the oscillations of the magnetic cantilever. Previous studies established the formation of a stripe maze pattern and a skyrmion crystal in CrBr_3_ with the length scale of the characteristic magnetic features dependent on the thickness of the film.^[^ ^6^ ^]^ In the bulk regime, the feature size followed Kittel's low, while in the atomically thin regime, the magnetic order collapsed in favor of large‐scale disordered domains.^[^ ^6^, ^7^, ^8^ ^]^ These findings are typical of ferromagnets exhibiting out‐of‐plane easy axis of magnetization within diverse crystal structures and are captured by traditional magnetic phase diagrams.^[^ ^9^ ^]^ Herewith, we demonstrated that in the intermediate regime of thicknesses between 175 and 300 nm, an unconventional magnetic order occurred intrinsically in exfoliated CrBr_3_ films, observable commonly across multiple samples as overlaying magnetic sub‐systems exhibiting distinct magnetization textures. Examples of two CrBr_3_ samples of 190 and 300 nm thickness hosting co‐existing magnetic sub‐orders are demonstrated in **Figure** **1** (see also Figure S1, Supporting Information). Although the magnetization patterns in each sample appear unique, they share common characteristics. Isolating periodic features in the MFM images via fast Fourier transform (FFT) (the procedure is described in Figure S7, Supporting Information) typically reveals an ordered magnetic sub‐system with smaller feature size characteristic of bulk CrBr_3_ films (Figure 1b,e) and a disordered magnetic sub‐system with significantly enhanced lateral extension of the domains characteristic of atomically thin CrBr_3_ films (Figure 1c,f). We attribute the emergence of these magnetic sub‐orders to stacking faults leading to the modifications of the interlayer exchange coupling constants. Stacking faults have been observed directly (e.g., via transmission electron microscopy^[^ ^10^ ^]^) and indirectly (e.g., via tunneling magnetoresistance^[^ ^11^ ^]^ or optically inspected ferromagnetic hysteresis loops^[^ ^12^, ^13^ ^]^) in layers of CrX_3_ crystals. Although it has been demonstrated that the modified arrangement of layers impacts the magnetic interactions and, consequently, plays a pivotal role in understanding and utilizing the optoelectronic properties of van der Waals ferromagnets,^[^ ^11^, ^14^, ^15^, ^16^, ^17^ ^]^ so far it has been unclear how the magnetic order is affected. We address this challenge by constructing a magnetic phase diagram that captures the existence and characteristics of the magnetic phases unique to van der Waals systems and two‐dimensional ferromagnetism.

### 2.2 Multi‐Domain Magnetic Phase Diagram

The origin of the commonly observed stacking faults can be elucidated by the first principle calculations.^[^ ^11^, ^18^, ^19^, ^20^ ^]^ Based on the density functional theory, we have calculated the total formation energy of a CrBr_3_ bilayer considering AA, AB, and M stacking configurations demonstrated in Figure S2, Supporting Information. Although AB stacking emerges as the ground state from the point of view of the stability of the crystal structure, the difference in the formation energy between the three configurations is minuscule. Therefore, in realistic samples at finite temperatures, the structural characteristics of CrBr_3_ crystals are based on a quasi‐degenerate energy landscape, where small energetic perturbations like heating or mechanical deformations are likely to cause the creation of stacking faults. Interestingly, the three stacking configurations differ dramatically in terms of interlayer exchange coupling constants: the AB and M stacking lead to ferromagnetic and antiferromagnetic exchange coupling, respectively, while AA stacking is characterized by an exchange coupling constant over two orders of magnitude weaker than in the other two cases. Consequently, the stacking faults in CrBr_3_ exhibit the potential to significantly modulate the strength and character (sign) of the interlayer exchange coupling.

**Figure 2:**
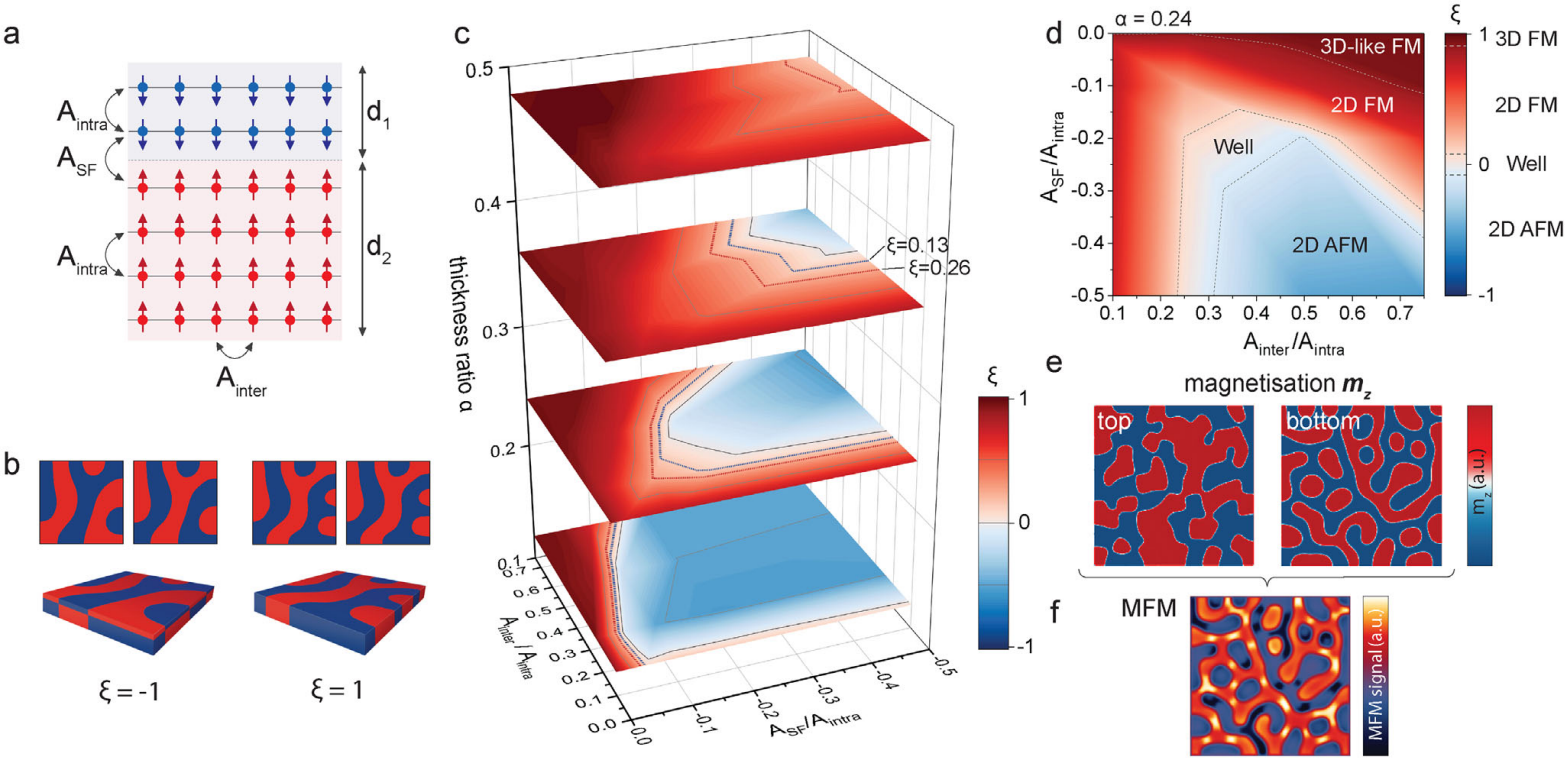
Influence of interlayer magnetic coupling on the domain structure. a) Schematic illustration of magnetic moments' arrangements in the presence of one stacking fault, introducing antiferromagnetic coupling between two groups of layers. b) Example of correlations between the sub‐domain patterns, where the similarity index ξ = 1 corresponds to identical domain patterns and ξ =–1 corresponds to inverted patterns. c) Phase diagram of the similarity index considering interlayer exchange stiffness, antiferromagnetic exchange between two groups of layers, and thickness ratio of the thinnest region $\alpha =\frac{d_{1}}{d_{1}+d_{2}}$ for a 50 nm thick sample. *A*_*inter*_ and *A*_*SF*_ are plotted in the values of *A*_*intra*_, representing the intralayer coupling. Dashed lines correspond to ξ = 0.26 (dark red) and 0.13 (dark blue) isopleths marking positions of the MFM‐measured samples shown in Figure 1 under the assumption of a minimal model with a singular stacking fault. d) Phase diagram of different magnetic configurations for the thickness ratio α = 0.24. e) Simulated magnetization for the top and bottom layers with ξ = –0.2, assuming *A*_*inter*_ = 0.5*A*_*intra*_, *A*_*SF*_ = − 0.5*A*_*intra*_, α = 0.24. f) Corresponding simulated MFM image for (e).

These considerations enable us to formulate a minimal model to account for the experimentally observed multi‐domain magnetization sub‐systems in CrBr_3_ crystals. We assume that the bulk layer is split by a stacking fault into two films characterized by thicknesses d_1_ and d_2_, describable by the thickness ratio α = d_1_/(d_1_ + d_2_), as shown in **Figure** **2**a. Separately, these two films exhibit magnetic characteristics typical of pristine CrBr_3_ crystal determined by the ferromagnetic intralayer exchange coupling (A_intra_), ferromagnetic interlayer exchange coupling (A_inter_), dipolar interaction, and single‐ion anisotropy. We introduced an interfacial exchange stiffness at the stacking fault (A_SF_), which coupled the two films together. The pristine CrBr_3_ crystal corresponds to the condition A_SF_ = A_inter_, while here we will focus on the case of antiferromagnetic coupling at the stacking fault (A_SF_ < 0) to consider the strongest possible perturbations to the magnetic order. Utilizing this model, we conducted micromagnetic simulations of the emergent magnetization textures exploring the dependence on α, A_inter_, and A_SF_, while assuming best‐known estimates for the remaining parameters (A_intra_, dipolar interaction, single‐ion anisotropy) characterizing the magnetic interactions. These calculations provide information on the spatial distribution of the out‐of‐plane magnetization (**M_z_**) extracted separately for both films (Figure 2e) and the stray magnetic field response of the entire structure (Figure 2f) directly comparable with the MFM images.

In order to establish a quantitative metric that captures the characteristics of the emergent magnetic order composed of coupled sub‐domain systems, we introduced a structural similarity index measure (SSIM) parameter, ξ, applied to two images representing the distribution of **M_z_** in the two films split by the stacking fault. The mathematical formulation of SSIM is given by:

(1) $$\xi \left(x,y\right)=\frac{\left(2\mu_{x}\mu_{y}+c_{1}\right)\left(2\sigma_{xy}+c_{2}\right)}{\left(\mu_{x}^{2}+\mu_{y}^{2}+c_{1}\right)\left(\sigma_{x}^{2}+\sigma_{y}^{2}+c_{2}\right)}$$

where µ_*x*_, µ_*x*_ are the means of the pixel intensity of images x and y, σ_*x*_, σ_*y*_ are the intensity variances of images x and y, σ_*xy*_ is the intensity covariance of images x and y, and *c*_1_, *c*_2_ are constant variables to stabilize dividing by a small denominator. In the physical picture, SSIM captures the difference in the total net magnetization in the two films and the degree of point‐by‐point variations between their respective magnetization textures. It is normalized to fulfill the condition: −1 ⩽ ξ ⩽ 1, where ξ = −1 corresponds to fully anticorrelated magnetic orders ($M_{z}^{\left(1\right)}=-M_{z}^{\left(2\right)}$ at each point) and ξ = 1 corresponds to fully correlated magnetic orders ($M_{z}^{\left(1\right)}=M_{z}^{\left(2\right)}$ at each point) as schematically illustrated in Figure 2b. ξ = 1 describes a traditional magnetic state occurring in three‐dimensional magnets characterized by a single magnetization texture extending in the entire bulk of the sample. The fully anticorrelated sub‐domains corresponding to ξ = −1 are not observable in the MFM images due to the compensation of the stray fields originating from the two magnetic sub‐systems. Our experimental results demonstrated that the typical magnetic order in CrBr_3_ films of intermediate thickness represents |ξ| ≪ 1 case.

The comparison between the MFM images and the results of the micromagnetic simulations points towards a conclusion that ξ may be considered a critical parameter determining the magnetic order in layers of CrBr_3_. Consequently, we can construct a phase diagram based on the micromagnetic simulations to describe the evolution of the magnetic order with the structure of the film (the position of the stacking fault given by α) and magnetic interactions (A_inter_, A_SF_). Such three‐dimensional phase diagram ξ(α, A_inter_, A_SF_) is presented in Figure 2c. It demonstrates that the emergence of vertical domain sub‐systems requires the stacking fault to isolate a thin magnetic layer from the bulk crystal consistently with the conclusions from the experimental MFM images. A decreasing α leads to the correlated sub‐domains emerging in a broader range of exchange coupling parameters and occupying a larger portion of the phase diagram. This observation correlates with the features of the magnetic order observed via MFM imaging. The isopleths of the experimental SSIM values of the two CrBr_3_ films presented in Figure 1 are demonstrated in Figure 2c. The experimental isopleths indicate that the stacking faults must be located close to the surface of the sample for the correlated sub‐orders to emerge in the realistic range of exchange coupling parameters. However, it is important to note that our model is based on minimal assumptions, which are sufficient to explain the microscopic origin of the magnetic sub‐systems. The experimental situation may be more complex, including cases of multiple stacking faults occurring at different positions in the sample, which is plausible on account of a large diversity of the magnetic orders observed in CrBr_3_ films as summarized in the Supporting Information Appendix in Figures S1,S4–S6, Supporting Information.

We identified four distinct types of magnetic order illustrated in the cross‐sectional phase diagram ξ(α = 0.24, A_inter_, A_SF_) in Figure 2d. Three‐dimensional ferromagnetism (3D FM) corresponds to a case in which the entire sample behaves as a monolithic system with a singular magnetic order despite the presence of a stacking fault with interfacial antiferromagnetic coupling. This state emerges in a regime of interactions fulfilling a condition |A_SF_| ≪ |A_inter_| when the dipolar interaction overcomes the weak exchange coupling at the interface rendering the stacking fault ineffective as a perturbation to the magnetic order. Two‐dimensional ferromagnetism (2D FM) is distinguished by the existence of a two‐domain sub‐system when both films separated by the stacking fault host a distinct magnetic order. The emergent two magnetization textures exhibit a finite degree of positive correlation represented by ξ > 0 implying that long‐range dipolar coupling acting in combination with ferromagnetic A_inter_ interaction dominates over the interfacial antiferromagnetic coupling determined by A_SF_. The further increase in A_SF_ interaction strength leads to a compensation effect when the magnetic orders in the two sub‐systems are fully decoupled in terms of the geometry of the magnetization textures as determined by the condition ξ ≈ 0. We designate this state as a van der Waals ferromagnetic well indicating that a thin magnetic layer hosts a magnetization texture that is independent of the bulk magnetic environment. We envisage parallels to other well‐type structures that could be employed to localize magnetic (magnons, magnetic polarons, skyrmions) and electronic (electrons, holes, excitons) excitations/states within the ferromagnetic well. Finally, in the regime that the A_SF_ dominates over other interactions in the magnetic system, anticorrelated magnetic orders begin to emerge characterized by ξ < 0 condition, labeled as a two‐dimensional antiferromagnetic state (2D AFM). However, with the increased degree of these anticorrelations, their detectability through MFM imaging becomes limited.

### 2.3 Influence of the Magnetic Field and Temperature on the Multi‐Domain Ferromagnetic System

**Figure 3:**
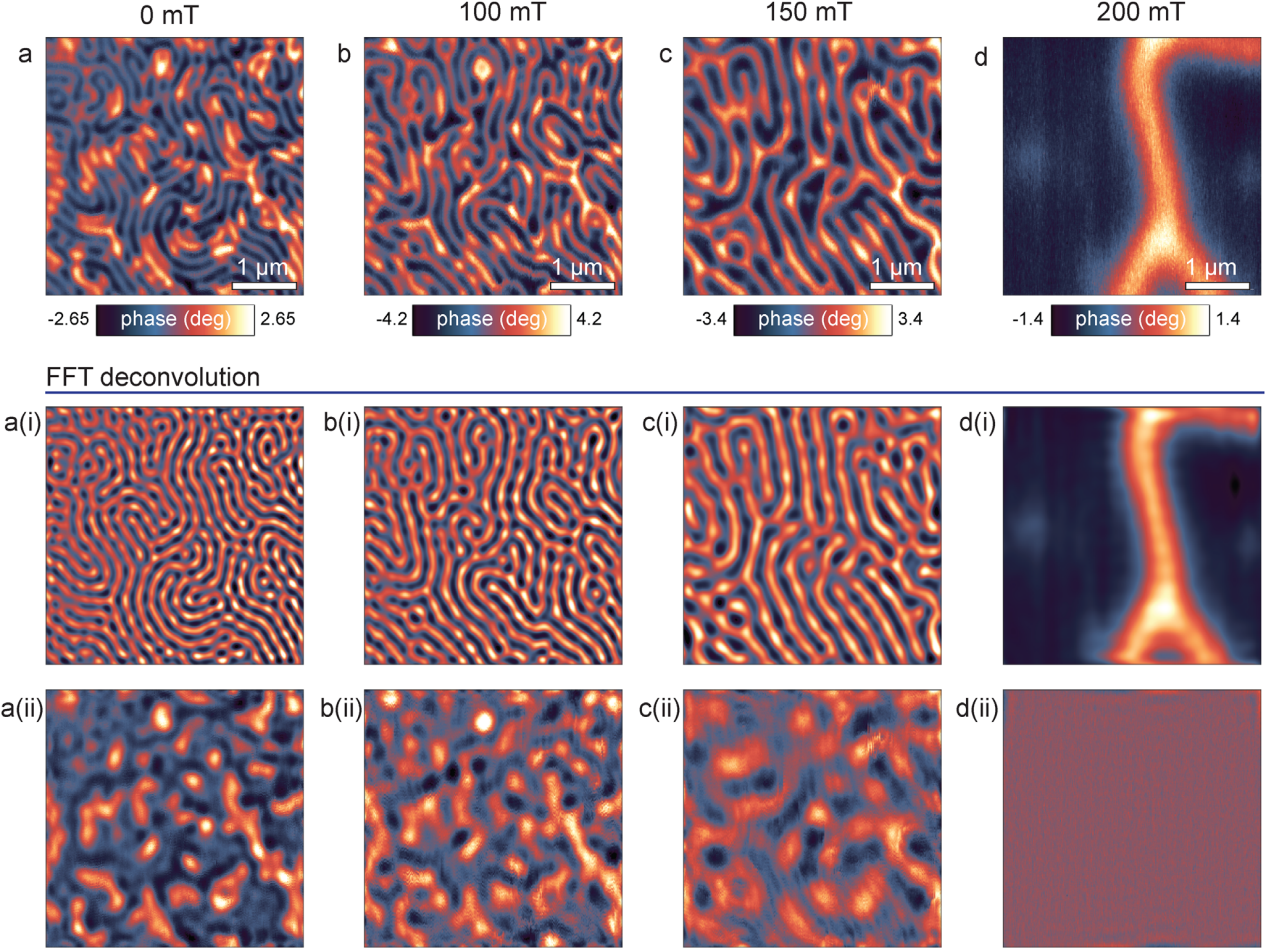
Evolution of two magnetic orders with the magnetic field. a–d) MFM maps measured at 0, 100, 150, and 200 mT at T = 1.7 K for 175 nm thick CrBr_3_ sample. Image (a) was deconvoluted into a(i) and a(ii) using FFT. Images (b–d) were deconvoluted in the same way.

A priori, multiple mechanisms can be utilized to traverse the multi‐domain magnetic phase diagram, as the magnetic interactions in van der Waals systems are tunable by an electric field, electrostatic and ionic doping,^[^ ^21^, ^22^, ^23^, ^24^, ^25^ ^]^ or pressure and strain.^[^ ^26^, ^27^, ^28^ ^]^ Here, we explore the effects induced by the application of a magnetic field and by varied temperature. The evolution of a two‐component magnetic order visualized via MFM imaging with an external magnetic field applied out‐of‐plane of the CrBr_3_ film is demonstrated in **Figure** **3** (see Figures S4–S6, Supporting Information, for other thicknesses). The impact of the magnetic field on the individual magnetic sub‐orders is unveiled by separating the periodic and disordered phases via a fast Fourier transform (Figure 3e–l). Both magnetization textures exhibit an enlargement of the size of the domain features with increasing magnetic field until saturation is reached. In agreement with previous results, the periodic stripe pattern attributed to the bulk CrBr_3_ film was characterized by a larger saturation field (above 200 mT) than the disordered pattern typical of atomically thin CrBr_3_ layers. However, the saturation field characterizing the disordered system was unusually high in the range between 150 and 200 mT, significantly exceeding the values of tens of mT typically observed for atomically thin CrBr_3_ films. This finding implies that despite relatively weak correlations in the geometry of the two magnetic orders, the magnetic interactions lead to the stabilization of the magnetic order in the thin film component at higher magnetic fields approaching those characteristic of bulk films.

**Figure 4:**
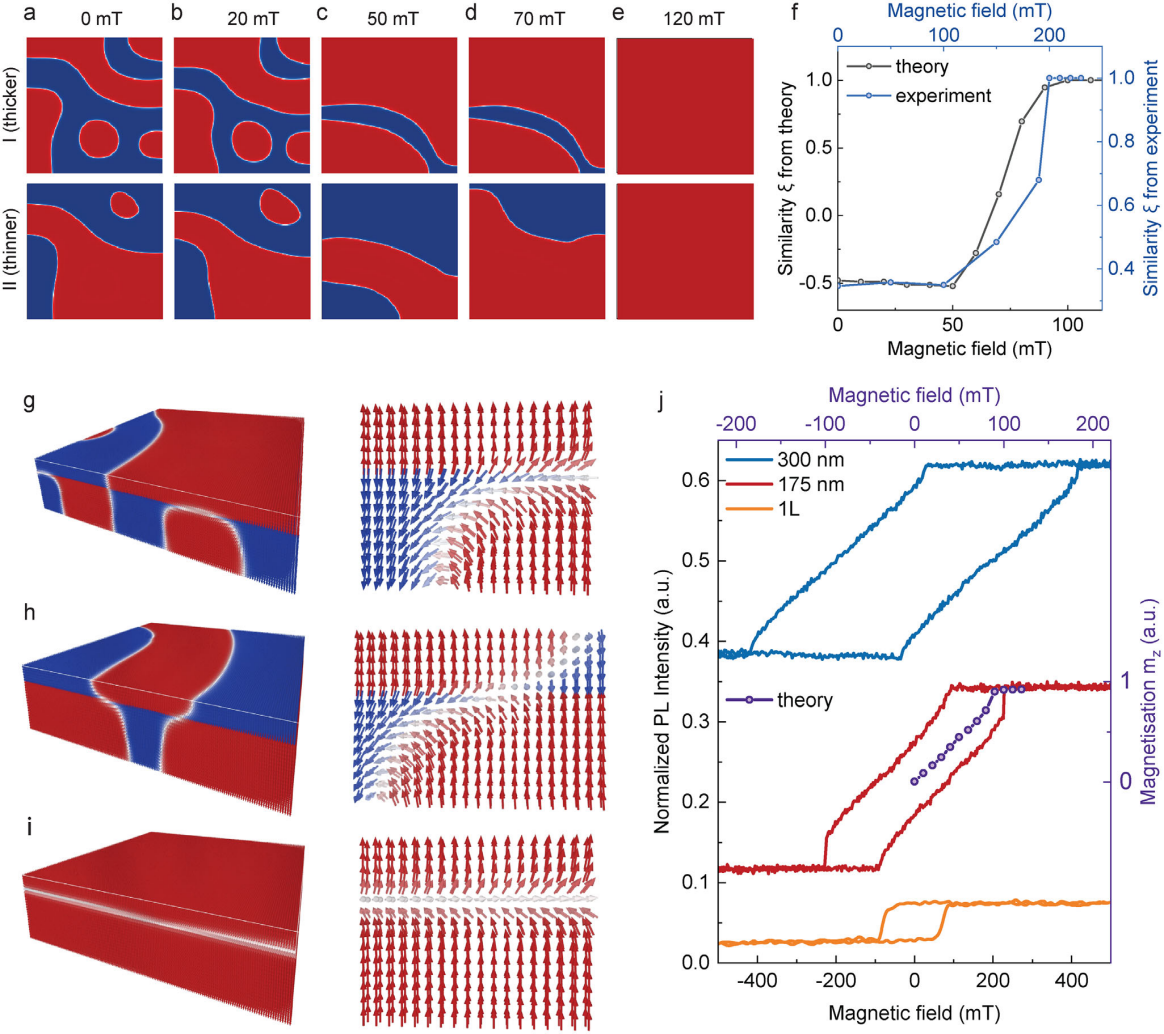
Micromagnetic simulations of the evolution of two magnetic orders with the magnetic field. a–e) Images of the out‐of‐plane magnetization in the bottom (thicker film) and the top (thinner film) layers at 0, 20, 50, 70, and 120 mT for 50 nm thick CrBr_3_ sample with thickness ratio α = 0.24, A_*inter*_=0.5A_*intra*_, A_*SF*_=‐0.4A_*intra*_. The image size is 400 × 400 nm. f) SSIM for theory (dark gray) and experiment (blue) as a function of a magnetic field. The experimental curve is calculated for the MFM images from Figure 3. g–i) Calculated domain configurations and spin arrangement near the stacking fault at different magnetic fields. j) Experimental magneto‐photoluminescence curves (lines) for 300 nm, 175 nm, and 1 layer thick CrBr_3_ samples and theoretical magnetization curve (violet line with circles) as a function of a magnetic field.

Micromagnetic simulations that account for the impact of an external magnetic field via introduction of a Zeeman term, corroborate such interpretation. The magnetic field evolution of the magnetization textures represented by the spatial distribution of M_z_ in two films isolated by a stacking fault in the regime of interactions that leads to a formation of weakly correlated magnetic sub‐orders (**Figure** **4**a–e and Figure S8, Supporting Information) exhibit an excellent agreement with the MFM imaging data. This agreement extends to the quantitative macroscale description of the magnetic order via similarity parameter, ξ, whose magnetic evolution is demonstrated in Figure 4f. ξ remains independent of the magnetic field until the Zeeman term becomes comparable with the interlayer exchange coupling A_SF_. At the magnetic field corresponding to this compensation, ξ starts to increase implying the reduction of the impact of the stacking fault on the magnetic sub‐orders. This leads to the conclusion that the magnetic field enables the modification of the magnetic order by the vertical traversing of the magnetic phase diagram presented in Figure 2d toward states exhibiting enhanced ferromagnetic character.

At a microscopic level, the evolution of the multi‐domain magnetic sub‐systems is driven by the behavior of the domain wall in the vicinity of the stacking fault as illustrated in Figures 4g–i. The micromagnetic simulations demonstrated that the increasing magnetic field facilitates the propagation of the domain wall across the stacking fault, which modifies the geometrical correlation between the two magnetic sub‐systems. The impact of the stacking fault is also pivotal in understanding the evolution of the total net magnetization of van der Waals ferromagnets in a general view. The collapse of a magnetic order reorients the domain wall to be aligned with the stacking fault as visualized in Figure 4i. This results in a sharp jump in the total net magnetization inspected as a function of the magnetic field, which can be reflected in the measurements of ferromagnetic hysteresis loops (e.g., via optical techniques, such as Kerr rotation or magnetic circular dichroism) or magnetoresistance in vertical tunneling junctions. We commonly observed sharp jumps in the hysteresis loops close to the saturation field inspected via measurements of the magnetic field evolution of the photoluminescence intensity from the CrBr_3_ films hosting multi‐domain sub‐systems in an intermediate thickness regime, as exemplified in Figure 4j. The rapid increase of the photoluminescence intensity correlates well with the jump in the total net magnetization obtained from the simulations.

The similarity index parameter, ξ, is also affected by the changes in the temperature (Figure S9, Supporting Information). The experimental data demonstrated an initial decrease of ξ with increasing temperature until the trend was reversed when approaching the critical Curie temperature. The analysis of the MFM images points toward a synergistic effect of the modification of the geometrical variations between the two magnetic sub‐orders in conjunction with changes in the relative contribution of the two systems to the total net magnetization. The micromagnetic simulations can predict the temperature evolution of the magnetic sub‐orders by assuming temperature‐dependent parameters describing the magnetic interactions according to the formulas presented in the Experimental Section. The results of these calculations (Figure S10, Supporting Information) reveal that the temperature evolution of the similarity index parameter, ξ, may be qualitatively different depending on the strength of the interfacial antiferromagnetic exchange A_SF_ relative to the pristine interlayer exchange coupling, A_inter_. Our findings demonstrate that the detailed evolution of the magnetic order with a magnetic field and temperature may exhibit a variety of diverse behaviors due to the structural characteristics of the CrBr_3_ driven by different types and numbers of stacking faults emerging in realistic samples.

## 3 Conclusion

We demonstrated that layers of van der Waals ferromagnet CrBr_3_ host an unconventional type of magnetic order consisting of vertically correlated magnetic sub‐domains. These sub‐systems originate from highly anisotropic exchange coupling constants and quasi‐degenerate structural energetics that enable the occurrence of stacking faults with largely modified interlayer magnetic interactions. A macroscopic measure of correlations in the form of a structural similarity index, indicative of the geometrical variations between the two magnetic sub‐orders, constitutes a basis for a magnetic phase diagram unique to van der Waals ferromagnets. Imaging the domains via magnetic force microscopy and the observation of the modified hysteresis loops via photoluminescence spectroscopy in conjunction with micromagnetic simulations leads to the conclusion that microscopic and macroscopic description of the coexisting magnetic orders is essential for the understanding of the properties of ferromagnetic films, heterostructures, and devices. This study opens questions regarding further developments of control knobs enabling desired degrees of correlations between magnetic sub‐systems and the broader impact of the existence of multi‐component magnetic orders for fundamental excitations in condensed matter physics.

## 4 Experimental Section

### Sample Fabrication

The CrBr_3_ crystal was purchased from HQ graphene. Thin CrBr_3_ layers were mechanically exfoliated on polydimethylsiloxane (PDMS) substrates in an inert gas glovebox with a water and oxygen concentration of less than 0.5 ppm. It was verified via the Raman scattering spectroscopy that the vibrational response of the exfoliated films was in agreement with the pristine CrBr_3_ crystal structure.^[^ ^29^ ^]^ Then a thin CrBr_3_ flake was transferred to a prepatterned gold electrode at room temperature that was made to ground the sample to avoid accumulation of electrostatic charge. Samples with thicknesses less than 100 nm were additionally encapsulated with hBN. The thicknesses of the CrBr_3_ flakes were first identified by optical contrast and then measured more precisely with an atomic force microscope. More details on sample preparation, protection from degradation,^[^ ^30^ ^]^ and sample analysis can be found in the Supporting Information and in methods in ref. [6].

### Magnetic Force Microscopy

Atomic force microscopy and MFM were performed using an attocube attoDRY 2100 closed‐cycle cryogenic microscope with a base temperature of 1.7 K, equipped with a superconducting magnet up to 9 T. Silicon probes with magnetic CoCr‐coating and with *k* of 2.5–5 N m^−1^ were used (Nanosensors SSS‐MFMR for higher resolution and PPP‐MFMR for higher sensitivity, NanoWorld MFMR and MFMR‐LM with lower magnetic moment). Before the measurements, the probes were magnetized at room temperature using a neodymium magnet. Magnetic contrast was observed in the phase signal in both tapping and non‐contact lift modes during the measurements. In the non‐contact regime, the cantilever was maintained at a constant lift height between 20 and 300 nm, depending on the sample thickness and its interaction with the domains. The lift height was selected to optimize sensitivity while minimizing domain dragging caused by the MFM tip. For samples thinner than 100 nm, the lift height was typically 20–30 nm, whereas for thicker samples, it ranged from 50 to 80 nm. Near magnetic saturation, the lift height was increased to up to 300 nm to further reduce dragging effects. During the measurements, the magnetic field was applied in an out‐of‐plane direction. Topography was additionally measured at elevated temperatures to decouple the magnetic signal from the surface morphology.

### Photoluminescence Measurements

Photoluminescence experiments were measured in a backscattering microscopic configuration in the same dry cryogenic system at 1.7 K. Samples were mounted on piezoelectric stages for precise *x*–*y*–*z* positioning. Continuous wave excitation at 730 nm was used, with a laser power of ≈50 µW. The laser light was focused using a lens with a numerical aperture of 0.82, yielding a spot of ≈1 µm in diameter. The light emitted from the sample was collimated by the same objective and scattered by a 0.75 m spectrometer equipped with a 150 lines mm^−1^ grating and a charge‐coupled device camera. The magnetic field was applied to the sample in the out‐of‐plane direction through superconducting coils. In magneto‐photoluminescence experiments, circular polarizations of the absorption was controlled using a polarizer and a λ/4 wave plate placed in the excitation path.

### Micromagnetic Simulations

To support the experimental results, micromagnetic simulations of CrBr_3_ magnetic ground states were performed using MuMax3.^[^ ^31^ ^]^ The value for saturation magnetization was set to *M*_*s*_ = 270 kA m^−1^, in accordance with measured values at low temperature.^[^ ^32^ ^]^ The uniaxial magnetic anisotropy constant was assumed to be *K*_*u*_ = 86  kJ m^−3^ along the out‐of‐plane direction, as has been reported for bulk material.^[^ ^32^ ^]^ The global exchange stiffness was estimated from the exponential rise of domain size to be *A*_*intra*_ = 10^−12^ J m^−1^.^[^ ^6^ ^]^

The simulation incorporated the experimentally measured thermal variation of *M*_*s*_(*T*) based on data from ref. [32] and equation *M*_*s*_(*T*) = *M*(0)(1 − (*T*/*T*_*c*_)^3/2^). The values for *K*_*u*_(*T*) were derived from equation [*K*_*u*_/*K*_*u*_(*T*)] = [*M*_*s*_/*M*_*s*_(*T*)]^3^, which forecasts a rapid decline in uniaxial magnetocrystalline anisotropy as the temperature increases. The thermal variations of *A*_*ex*_ were approximated using their mean field approaches, suggesting a nearly square dependency [*M*_*s*_/*M*_*s*_(*T*)]^2^.^[^ ^33^, ^34^ ^]^

To take into account weaker ferromagnetic interlayer coupling, the simulated space was divided into separated layered regions with rescaled exchange stiffness *A*_*inter*_. To simulate the AFM coupling that occurred due to different stacking, the exchange stiffness *A*_*SF*_ was set to a negative value between certain layers depending on the set thickness ratio of the two regions. Inter‐ and intralayer exchange constants were selected based on the DFT calculation. However, as DFT calculation typically exhibits limited accuracy, a broader range of parameters was considered to ensure that the phase diagram captures interactions occurring in realistic samples.

To identify the degree of similarity of two domain patterns of neighbouring domain groups, the structural similarity index measure (SSIM)^[^ ^35^ ^]^ ξ was used. SSIM was equal to 1 for completely the same images and –1 for the case of very dissimilar images, which can be the case for fully or partially inverted images and can give information on antiferromagnetic ordering between layers. For the case of different patterns, ξ = 0. After running simulations, out‐of‐plane magnetization *m*_*z*_ black‐and‐white maps (black is for spins pointing down, white is for spins up) for layers from two different domain groups separated with the antiferromagnetic interface were compared, and SSIM was calculated for them. SSIM phase diagram was obtained for the simulated images at 400 × 400 nm^2^ for the sample with a thickness of 50 nm. The result of SSIM can be sensitive to the size of the sliding window used for the comparison, which was set to the default value of 11×11 while calculating the phase diagram. The stabilization constants *c*_1_ = *K*_1_ *L* and *c*_2_ = *K*_2_ *L* of the formula 1 were also set to default values, where *L* is the range of the pixel values, *K*_1_ = 0.01 and *K*_2_ = 0.03.

In general, SSIM was based on three components when comparing two images: contrast, luminescence, and structure. The mean intensity of the signals determined the luminescence between them. The standard deviation defined the contrast. The structural component was defined by the correlation of the two images.

It is worth mentioning that other similarity metrics can also be implemented instead (see Figure S15, Supporting Information). One of the conditions which should be followed is distinguishing between the same and inverted images; otherwise, cases with antiferromagnetic coupling will be completely lost. Another feature that should be analyzed is structural information, as this is the key point for analyzing domain patterns from different groups.

### DFT Calculations

To calculate the energies of CrBr_3_ structures with different stacking, density functional theory available in the VASP code was used.^[^ ^36^ ^]^ Projector‐augmented wave pseudopotentials within the PBE parametrization were used together with 600 eV energy cutoff, 10 meV smearing of electronic occupations and 20×20×10 k‐mesh to sample the Brillouin zone. Description of electronic correlations was improved, compared to pure DFT, using the DFT+U scheme^[^ ^37^ ^]^ where U = 3 eV and J_H_ = 0.96 eV are defined for the 3d states of magnetic Cr. In that case, stable Cr moments of 3.0 µ_B_ and electronic bandgap of around 2.2 eV were obtained. The crystal structure, i.e., lattice vectors and atomic positions, was fully optimized until the mechanical stress and ionic forces were close to zero. This was done separately for ferromagnetic and antiferromagnetic configurations where the Cr spins were either parallel in all CrBr_3_ layers or antiparallel in the neighboring layers.

For the optimized structure, the Heisenberg magnetic interactions were calculated using the magnetic force theorem within the LKAG approach,^[^ ^38^ ^]^ which is a well‐established and computationally efficient approach available in the RSPt software^[^ ^39^, ^40^ ^]^ (see review^[^ ^41^ ^]^ and recent example studies^[^ ^42^, ^43^ ^]^). In these calculations, the information about interactions for different spin neighbors within a certain real‐space cutoff radius was obtained from a calculation done on a primitive cell, while the Brillouin zone was sampled using a fine k‐mesh with 1000 points in this case. This k‐point resolution was sufficient for calculating the first several nearest‐neighbor magnetic interaction parameters that were relevant for the discussion of ferro‐ and antiferromagnetic couplings of different CrBr_3_ domains in the main text.

## Conflict of Interest

The authors declare no conflict of interest.

## Author Contributions

S.Y.G., M.K., and K.S.N. conceived the project. S.Y.G. performed low‐temperature MFM experiments and analyzed the data. M.G. conducted magneto‐photoluminescence and Raman spectroscopy measurements. Z.C. and M.S. fabricated the samples. S.Y.G. performed the micromagnetic simulations with support from V.B. V.B., M.P., O.E., and M.I.K. conducted DFT calculations and provided theoretical support. S.Y.G. and M.K. wrote the manuscript with inputs from all co‐authors. All co‐authors contributed to this work, read the manuscript, discussed the results, and agreed on the included contents.

## Supporting information

Supporting Information

## Data Availability

The data that support the findings of this study are available from the corresponding author upon reasonable request.
