# Supplementary material for: Correlations in Magnetic Sub‐Domains as an Unconventional Phase Diagram for van der Waals Ferromagnets
Source: Adv Sci (Weinh). 2025 Apr 11;12(26):2500562. doi: 10.1002/advs.202500562 (PMC12245078; doi:10.1002/advs.202500562)
Supplement: Supplementary file 1 — Supporting Information [file ADVS-12-2500562-s001.pdf]

ADVANCED SCIENCE
Open Access

Supporting Information

for *Adv. Sci.*, DOI 10.1002/advs.202500562

Correlations in Magnetic Sub-Domains as an Unconventional Phase Diagram for van der Waals Ferromagnets

Sergey Y. Grebenchuk, Magdalena Grzeszczyk, Zhaolong Chen, Makars Šiškins, Vladislav Borisov, Manuel Pereiro, Mikhail I. Katsnelson, Olle Eriksson, Kostya S. Novoselov and Maciej Koperski**

Supplementary Information for: "Correlations in magnetic sub-domains as an unconventional phase diagram for van der Waals ferromagnets."

Sergey Y. Grebenchuk,[*,1,2] Magdalena Grzeszczyk,[1] Zhaolong Chen,[1,3] Makars Šiškins,[1] Vladislav Borisov,[4] Manuel Pereiro,[4] Mikhail Katsnelson,[5,6] Olle Eriksson,[4,7] Kostya S. Novoselov,[1,2] and Maciej Koperski[*,1,2]

[1]*Institute for Functional Intelligent Materials, National University of Singapore, 117544, Singapore*

[2]*Department of Materials Science and Engineering, National University of Singapore, 117575, Singapore*

[3]*School of Advanced Materials, Peking University Shenzhen Graduate School, Shenzhen, 518055, China*

[4]*Department of Physics and Astronomy, Uppsala University, Box 516, SE-75120 Uppsala, Sweden*

[5]*Institute for Molecules and Materials, Radboud University, Heyendaalseweg 135, NL-6525 AJ Nijmegen, Netherlands*

[6]*Wallenberg Initiative Materials Science for Sustainability, Department of Physics and Astronomy, Uppsala University, 75121 Uppsala, Sweden*

[7]*School of Science and Technology, Örebro University, SE-701 82 Örebro, Sweden*

* E-mail: sergey.gr.sc@gmail.com; msemaci@nus.edu.sg

Different cases of coexistence of domain patterns

Depending on the thickness of the sample, its quality, the number of stacking faults, and the history of the magnetic field applied, it is possible to get completely different cases of coexistence of the magnetic domains. Fig. S1 shows a few different cases obtained at different magnetic fields on 190 nm thick sample. Case 1 on Fig. S1 (a) demonstrates similar situation to Fig. 1 (a) of the main text, where two domain systems coexist at the same time. In Fig. S1 (b,c) more complicated picture is shown. There, at least three different domain systems are formed. In both scenarios, a crack-like feature appears upon sweeping the external magnetic field. Outside of this region, there are three domain subsystems: narrow stripe domains, domains with larger magnetization, which are mostly perpendicular to the small domains, and wavy wide domains, signals from which overlay the stripe domains. This third type of domain mostly follows the direction of the narrow stripe domains, creating hex-like shapes in some places. All the large varieties of magnetic ordering create complex patterns, like the shapes in Fig. S1 (c), which are similar to a spider web. Measurements in thicker samples also showed several domain patterns that existed simultaneously. For example, in a 300 nm thick sample, we also observed narrow stripe domains along with large disordered domains.

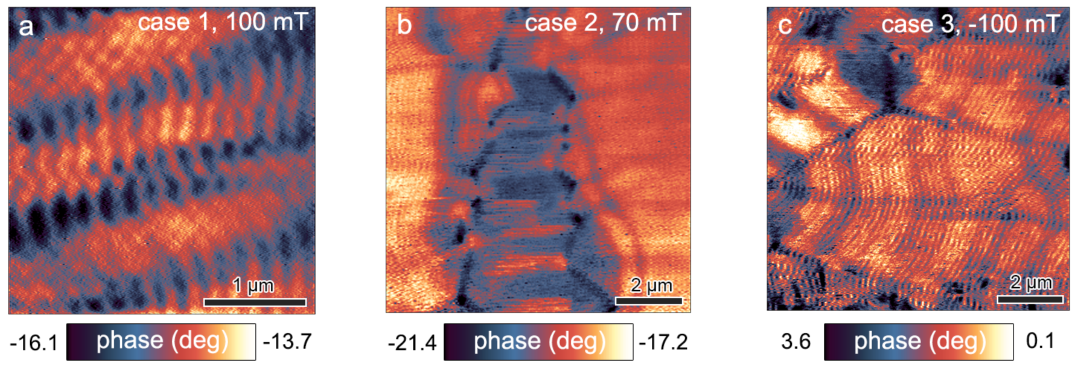

Figure S1: Coexistence for several domain subsystems for 190 nm thick $CrBr_3$ flakes. Three examples are taken at different magnetic fields and at different places. The lift height is 50 nm. On the MFM images, one can see at least two (a) or three (b,c) coexisting magnetic patterns of different intensities, shapes and sizes.

DFT calculations

Influence of stacking on interlayer magnetic coupling

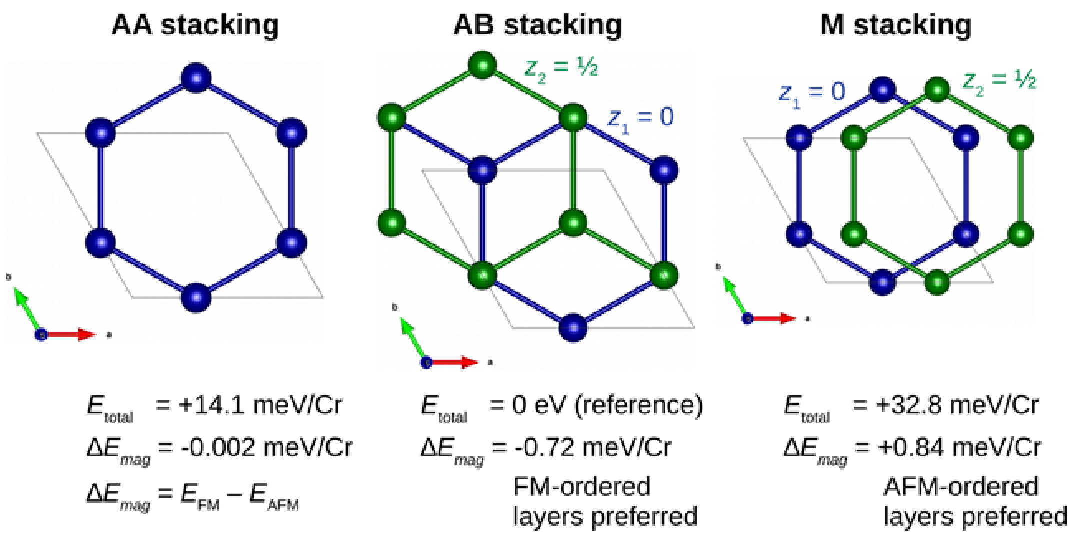

Figure S2: DFT calculations of energy and exchange stiffness for different stackings.

From the DFT calculations summarized in Figure S2, one can see that the AB stacking has the lowest energy. However, the other 2 stackings are not far away in terms of energy (within 33 meV per Cr atom), meaning that all 3 stacking types can, in principle, coexist in one sample, depending on the sample preparation conditions. One may also see that the relative magnetic ordering of different $CrBr_3$ layers is quite sensitive to the layer stacking, which is not surprising due to large differences in the relative shift of neighboring layers in the in-plane direction (compare the "green" and "blue" layers in Figure S2). Interestingly, in the AA-type structure the FM and AFM orderings have almost the same energy, due to compensation of Heisenberg interactions between different neighbors that can be of opposite signs.

Regarding the magnetic interactions, which we calculate using the LKAG approach[S1,S2] in the RSPt software,[S3,S4] we find that the interactions across the layers are much weaker (by 1-2 orders of magnitude) compared to the interactions within each layer. This is reflected in the theoretical estimates of the intra- and interlayer spin stiffness constants A_{xy} and A_z,

which are 0.37 pJ/m and 0.01 pJ/m for the AB stacking. These values are in line with the model parameters that we used for micromagnetic analysis in the main text.

Dependence of correlation phase diagram on the intralayer exchange stiffness

In addition to dependencies on interlayer exchange stiffnesses, a phase diagram was plotted for different intralayer exchange couplings A_{intra} (Fig. S3). Here, it can be seen that reducing A_{intra} leads to a shift of the region with $\xi < 0$ towards higher values of antiferromagnetic coupling A_{SF} and interlayer coupling A_{inter}.

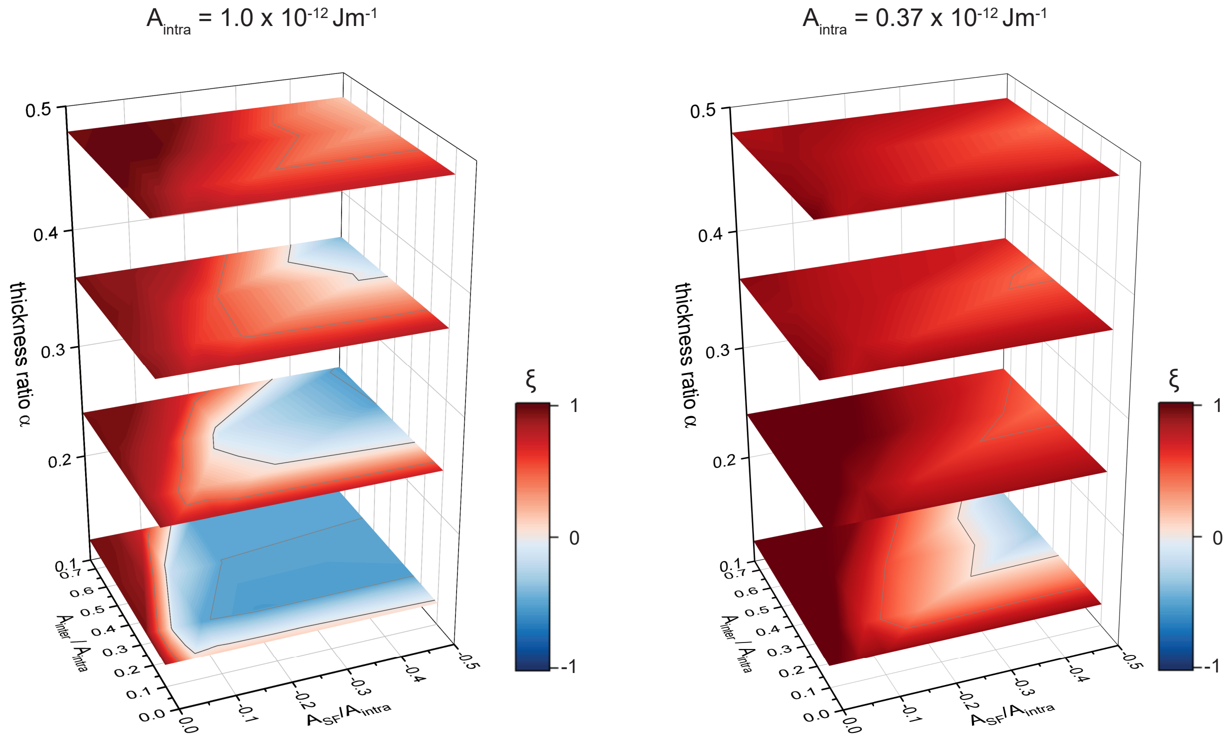

Figure S3: Correlation phase diagrams for different values of intralayer exchange stiffness A_{intra}.

MFM vs H for different thicknesses

Fig. S4 shows the evolution of the domain structure with sweeping of the external magnetic field. Figs. S4 (a-p) present the behavior of the magnetic ordering of a larger scale 20×20 μm^2. First, at 0 mT only large irregular magnetic domains are seen. On a smaller scale, there are also narrow periodic stripe domains around 90 nm in width. Then, at a higher magnetic field, two subsystems of striped domains appeared. The smaller domains are periodic and have a size of around 100 nm. At the same time, the magnetic signal from larger non-periodic domains can be seen. Moreover, these domains pattern is perpendicular to the direction of the smaller ones. Then, at higher magnetic fields larger domains disappear when narrow domains just broadened in size. It is worth mentioning that at higher magnetic fields close to saturation, the magnetic structure becomes less stable and can be dragged with the tip. For example, to decrease the influence of the cantilever and avoid dragging of the domains, the tip height was changed from 100 to 300 nm at H = 190 mT. Eventually, at 200 mT the saturation is reached. However, while the magnetic field decreases, an unusual crack-like feature appears. This structure has several things that warrant attention. Its borders consist of domains with stronger magnetization than other places, and along the border bubbles can be met. Inside the crack region domain pinning is the weak and magnetic structure can be modified by the MFM cantilever. It can be seen as dragging the domains in forward and backward MFM scans. Meanwhile, outside of the region, narrow stripe domains are present. The crack region grows with decreasing magnetic field and reaches its maximum size at 0 mT. Then, during increasing the magnetic field in the opposite direction, the crack feature actually starts to shrink and eventually completely disappears in the range of -100 and -130 mT, and only smaller stripe domains are left. Like previously, these narrow domains continue to increase in size upon increasing the magnetic field, then around -180 mT the ratio of domains oriented along the external field became bigger and continued to rise until saturation. Then the MFM scans were again measured in the range between -200 mT and 0 mT. It is quite peculiar that the crack-like feature was not only reproducible, but also emerged in a

different location. It leads to the thought that it is not directly related to topography or certain defects, but that there are also additional random factors that cause this feature to appear. In general, the system behaved similarly during the measurements from +200 mT to 0 mT. However, so far, domains inside of the region were more stable.

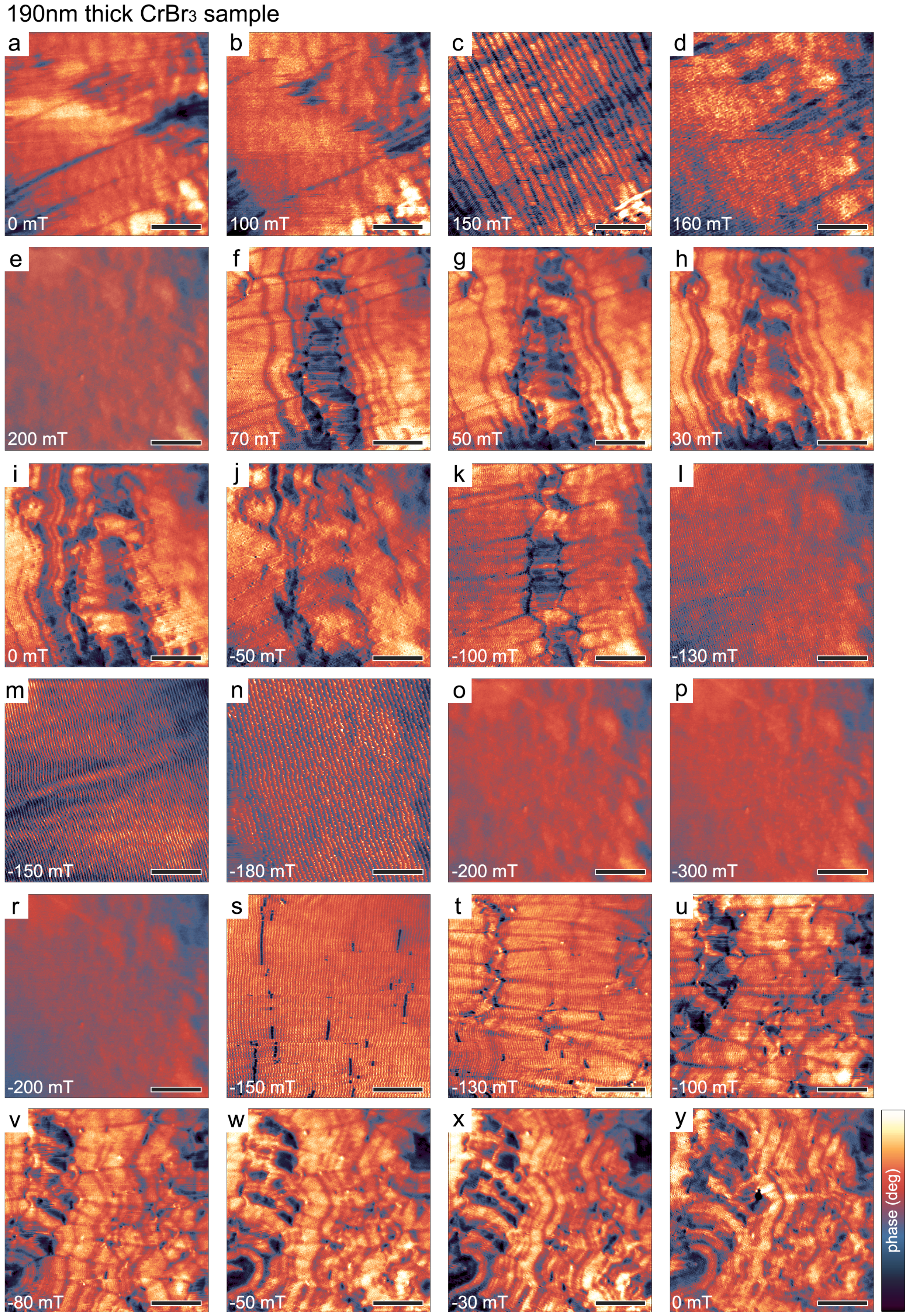

Figure S4: MFM scans obtained for 190 nm thick sample at different magnetic fields at T=1.67 K with lift height 100 nm. The scale bar corresponds to 5 μm.

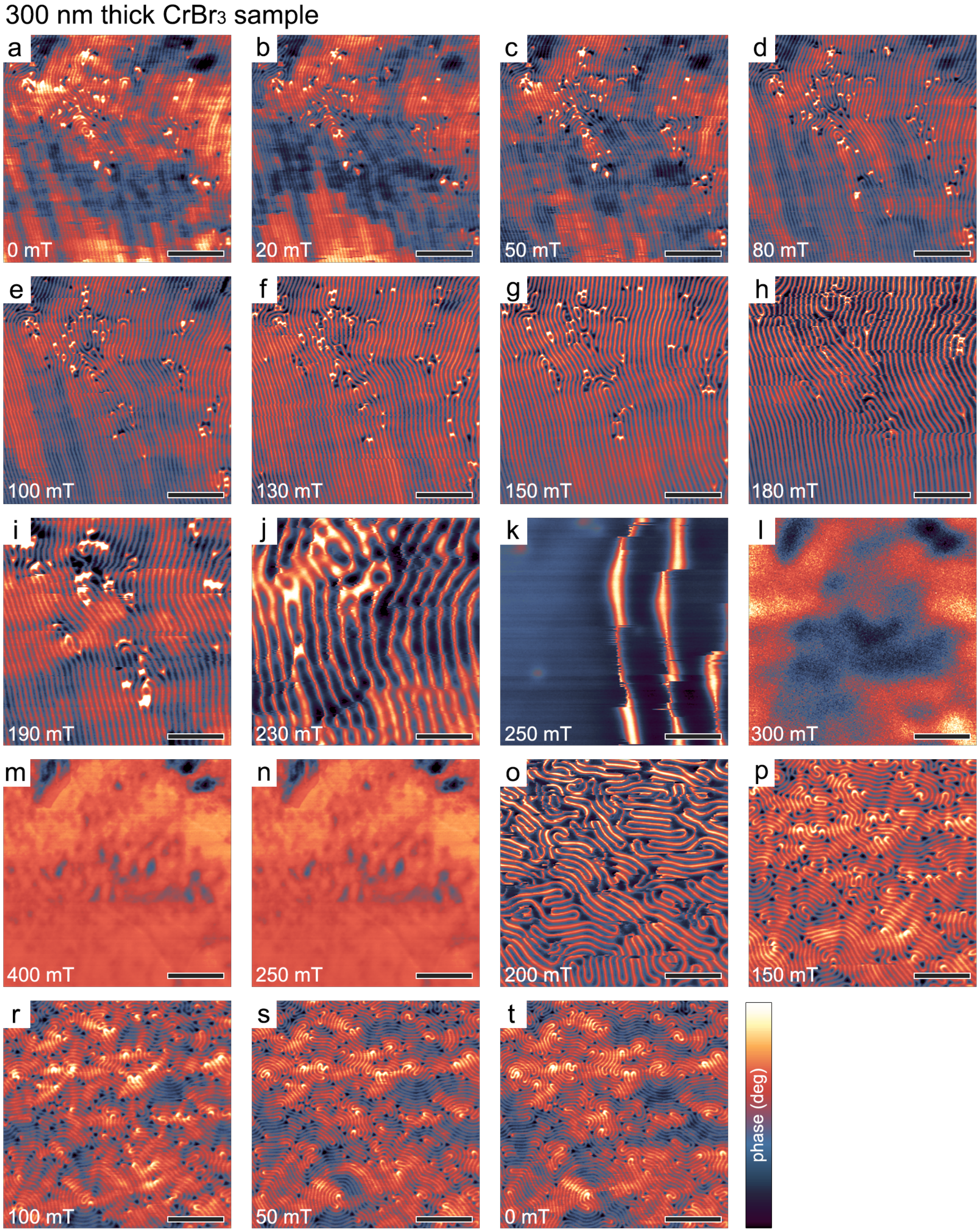

Figure S5: MFM scans obtained for 300 nm thick sample at different magnetic fields at T=1.67 K with lift height 100 nm. The scale bar corresponds to 5 μm.

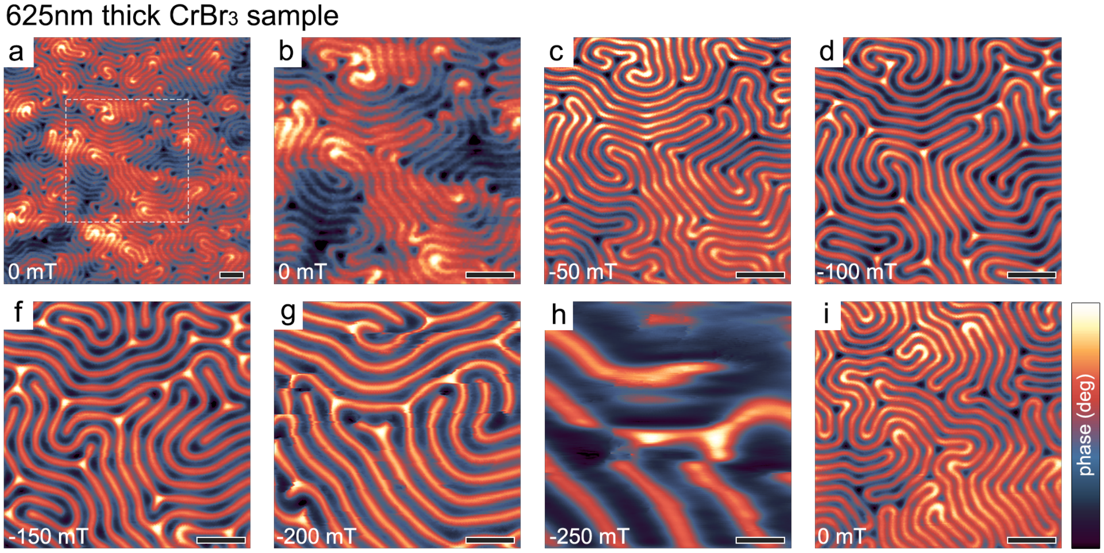

Figure S6: MFM scans obtained for 625 nm thick sample at different magnetic fields at T=1.67 K with lift height 100 nm. The scale bar corresponds to 2 μm.

Procedure for FFT deconvolution of MFM images

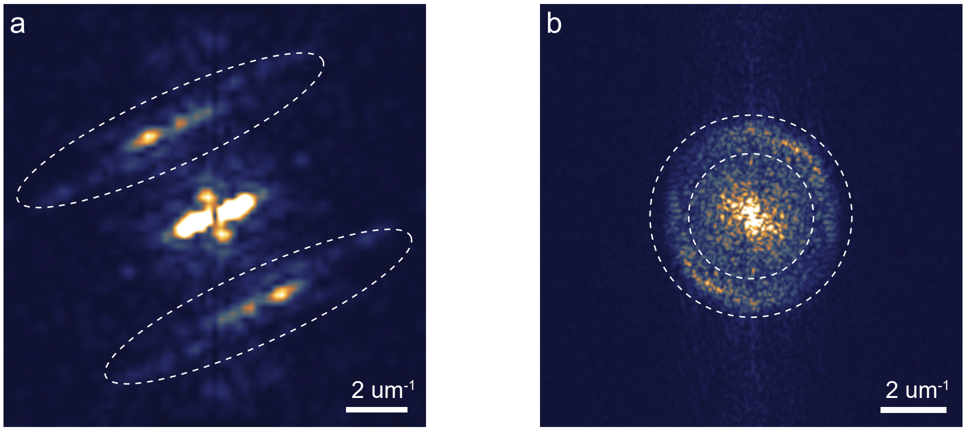

Figure S7: **FFT image deconvolution.** (a) - FFT for image Fig. 1 (a) of the main text, (b) - for Fig. 1 (d) of the main text. The regions within the area limited with dashed white lines were referred to the ordered domains, while the rest of the FFT region was considered to contribute to the second less ordered state.

Simulations of domains in the external magnetic field

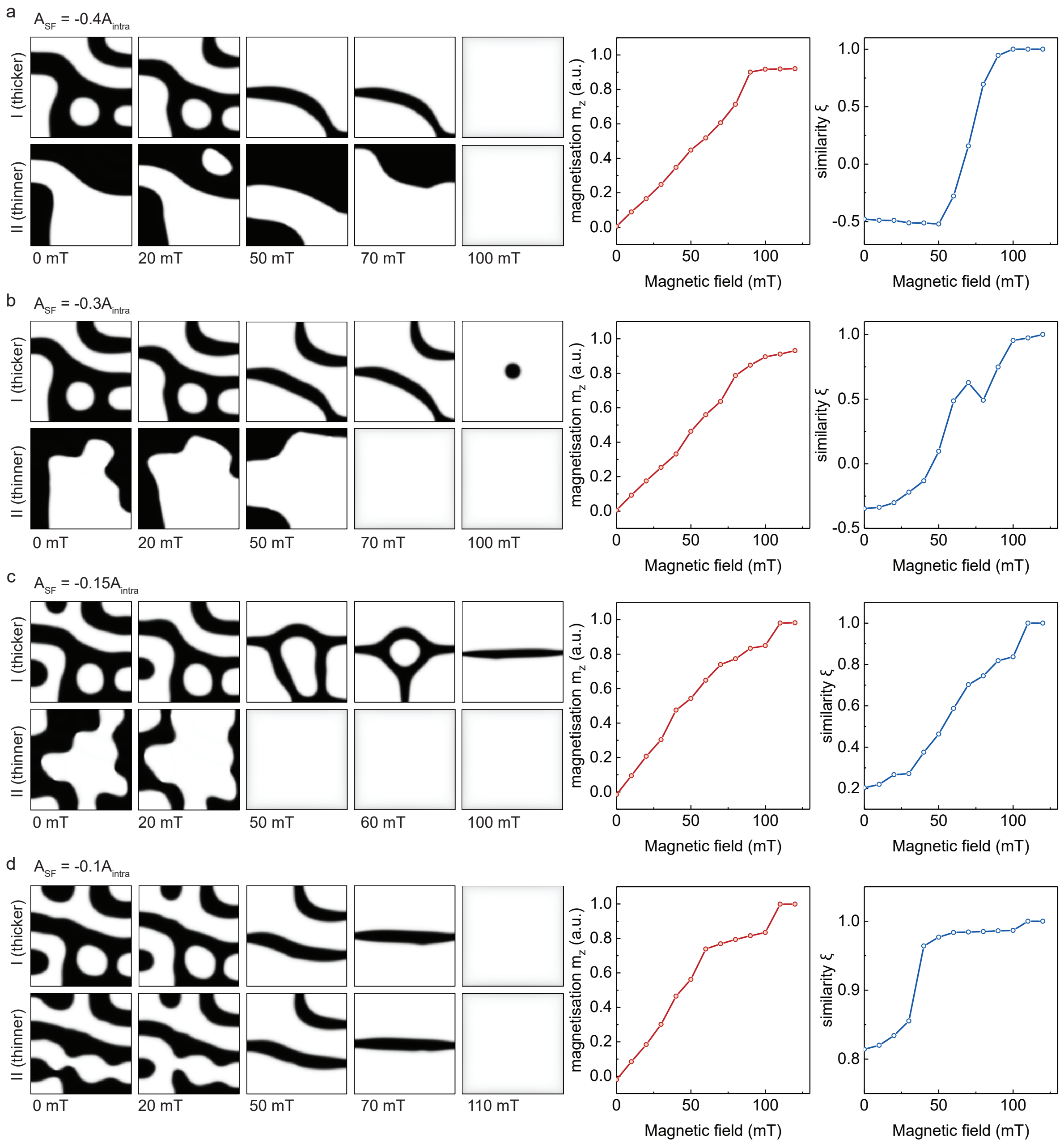

Figure S8: **Micromagnetic simulations of magnetic field dependence of magnetisation and SSIM.** (a-d) - micromagnetic simulations results for different exchange stiffness on the interface with antiferromagnetic coupling. Two rows represent z component of magnetisation of top and bottom layers of the 50 nm thick crystal at T = 0 K at different magnetic fields. Red and blue curves shows magnetisation and SSIM dependencies on the external out-of-plane magnetic field, correspondingly. Other parameters used for simulations: thickness ratio t = 0.24, $A_{inter} = 0.5A_{intra}$, the lateral image size is 400×400 nm^2.

Temperature dependence of the magnetization textures

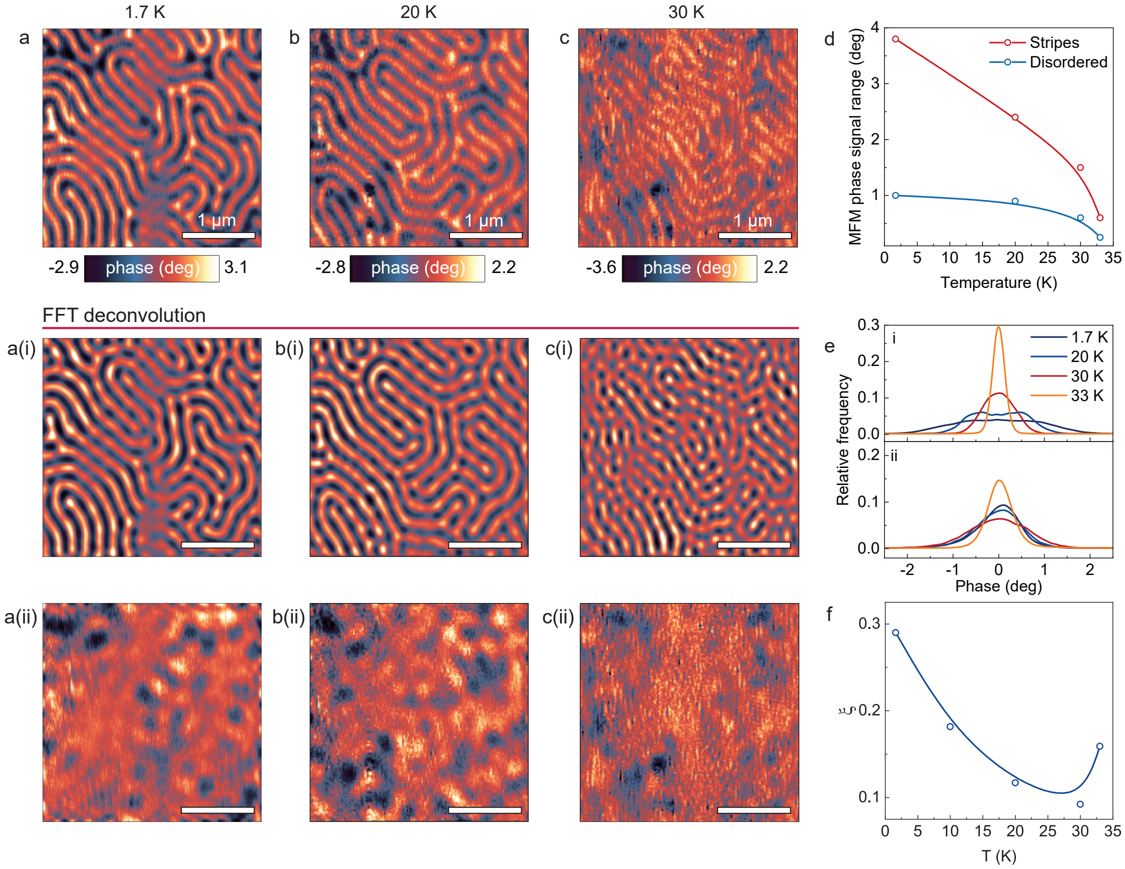

Figure S9: **Temperature dependence of two magnetic orders.** a-c - MFM maps measured at zero field at 1.7 K (a), 20 K (b), and 30 K (c) for 175 nm thick $CrBr_3$ sample. Image (a) was deconvoluted into a(i) and a(ii) using FFT. Images b-c were deconvolted in the same way. d - Dependence of the peak-to-peak signal of the domains on the temperature for both channels (i) with stripe domains and (ii) with disordered domains. e - Phase distributions of MFM maps at different temperatures for channels (i) and (ii). f - Dependence of the correlation parameter ξ on temperature.

Simulations of domains at different temperatures

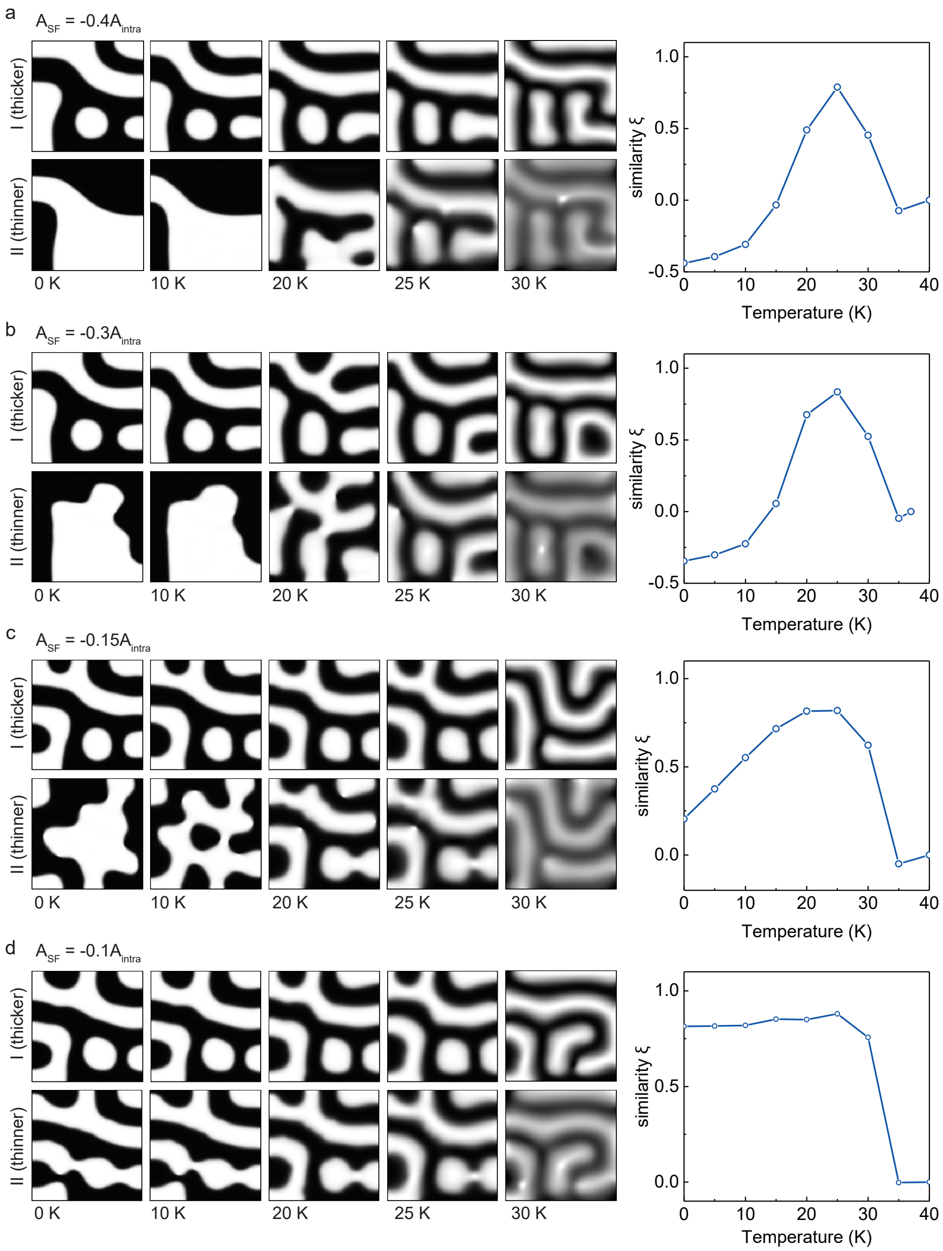

Figure S10: **Micromagnetic simulations of temperature dependence of magnetisation and SSIM.** (a-d) - micromagnetic simulations results for different exchange stiffness on the interface with antiferromagnetic coupling. Two rows represent z component of magnetisation of top and bottom layers of the 50 nm thick crystal at zero magnetic field at different temperatures. Red and blue curves shows magnetisation and SSIM dependencies on the temperature, correspondingly. Other parameters used for simulations: thickness ratio t = 0.24, $A_{inter} = 0.5A_{intra}$, the lateral image size is 400×400 nm^2.

The structural characterization of the $CrBr_3$ films.

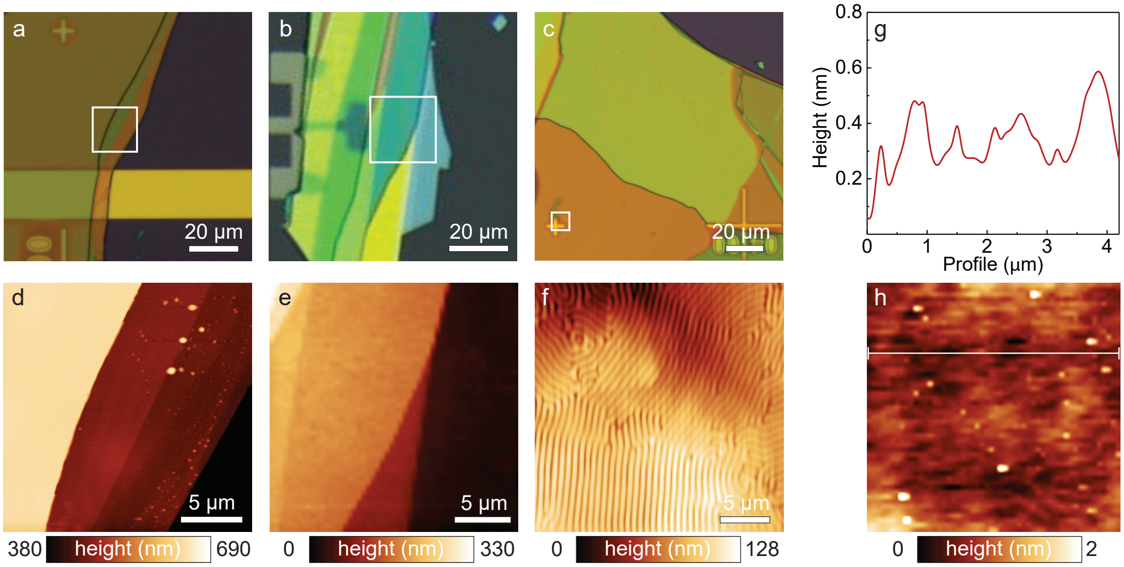

Figure S11: **Atomic force microscopy images of the studied flakes.** (a-c) - optical microscope images of the $CrBr_3$ flakes. (b-f) - Atomic force microscopy images from the areas highlighted with white rectangular in panels (a-c). (g) - Height profile taken along the white line from the height scan (h), demonstrating low level of roughness and atomically flat surface of the sample.

Fig. S11 presents surface characterization with atomic force microscopy of the studied samples. Fig. S11 (d,e) illustrates topographic images of the region on the flake with varying thicknesses, highlighting the presence of flat surface areas in the samples. Panel Fig. S11 (f) shows topography of the sample in the region where it is located on top of a gold marker (marked with a white square in Fig. S11 (c)). It can be seen that magnetic signal from the domains leaks into the height channel of the topography when measured in the tapping mode of the atomic force microscope, in which strong forces can interfere with the signal. Furthermore, the domains are not significantly influenced by slight deformation of the $CrBr_3$ film, as the magnetism of thicker samples is less susceptible to corrugation and roughness of the substrate surface. It is important to mention that this is not the case for very thin samples where even small defects can cause modification of magnetic properties and domain pinning.[S5] Fig S11 (g) shows a profile taken along the white line in the scan Fig. S11 (h),

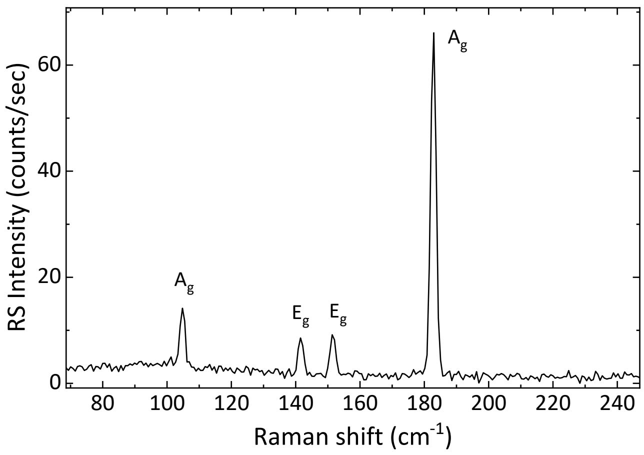

Figure S12: **Raman spectroscopy of $CrBr_3$ flakes.** Raman spectrum excited by a continuous wave laser at 2.33 eV at 1.7 K of 190 nm thick $CrBr_3$ flake showing no signs of mechanical deformation or strain present in the sample. The spectrum is measured on the area where MFM scans were performed which are presented in Fig. 1(a) of the main text and Fig. S1 and S4 in the SI.

demonstrating very small level of surface roughness that does not exceed 1 nm, indicating absence of significant deformation of the sample.

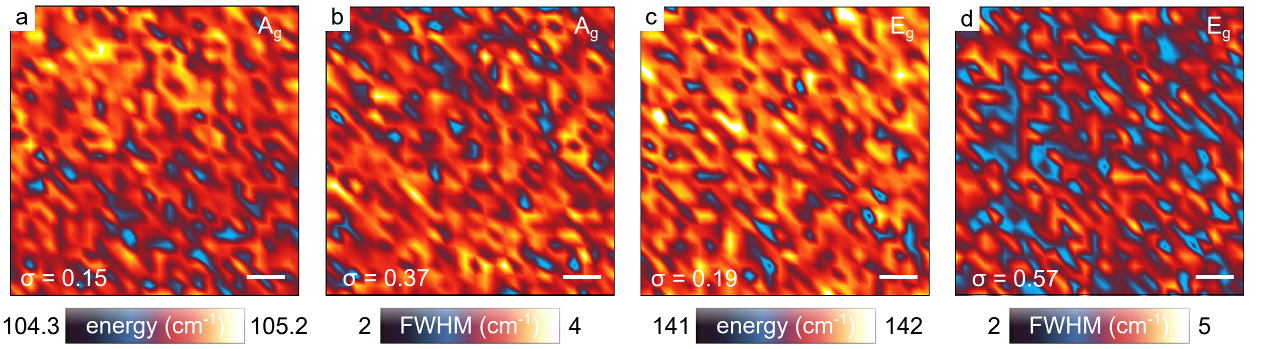

Figure S13: **Raman scattering map of $CrBr_3$ flakes.** Spatially resolved Raman maps of a 190 nm thick $CrBr_3$ flake at 1.7 K, excited by a continuous wave laser at 2.33 eV. (a) Energy of the A_g peak (b) its full width at half maximum (FWHM), (c) energy of the E_g peak, and (d) its FWHM. Standard deviations are included for each map to indicate measurement variations. The maps correspond to the same region where MFM scans were performed, as shown in Fig. 1(a) of the main text and Fig. S1 and S4 in the SI. The scale bar corresponds to 5 μm.

The typical Raman scattering spectra, shown in Fig. S12 align well with previously

reported results.[S6,S7] For further characterization of the above samples, we conducted spatial mapping of Raman scattering on the $CrBr_3$ flake (see Fig. S13). The investigated area was 38 μmm wide in both directions. Based on the maps, the standard deviation of the energy of the A_g and E_g modes does not exceed 0.19 cm^{-1}, indicating acceptable homogeneity of the studied sample. This suggests that the investigated flakes are relatively free of significant defects or strain-induced inhomogeneities. Furthermore, the distribution of the energy and FWHM is homogeneous across the studied area, with no discernible patterns or domain-like structures, further supporting the notion of a strain-free surface.

Mixed phase in thin $CrBr_3$ flakes

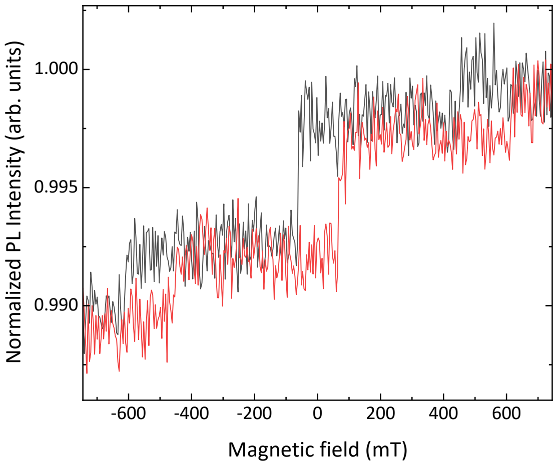

Figure S14: Magneto-photoluminescence hysteresis loop of $CrBr_3$ thin flake (≈50 nm) at 1.7 K, excited by a continuous wave laser at 1.698 eV, revealing characteristic contributions from both antiferromagnetic and ferromagnetic components.

Our magneto-photoluminescence hysteresis loop measured on a thin $CrBr_3$ flake exhibits a three-step transition with the magnetic field, similar to observations reported in Ref. S8. As in previous studies, we observe a residual ferromagnetic signal near zero field, followed by two successive spin-flip transitions at higher fields. This suggests a coexistence of FM and AFM components.

Comparison of different similarity metrics

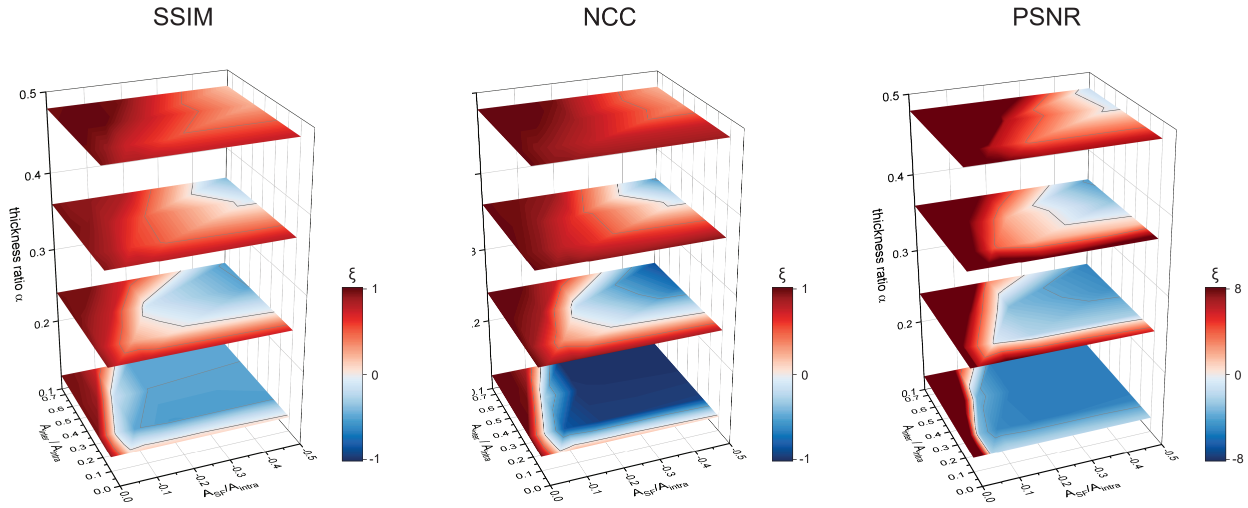

Figure S15: **Correlation phase diagrams for the case of using different metrics for the same set of simulated images.** (a) - Correlation phase diagram plotted using Structural Similarity Index Measure (SSIM), (b) - Normalized Cross-Correlation (NCC) metric, (c) - Peak Signal-to-Noise Ratio (PSNR) metric.

For the case of magnetization images, most of the area is has magnetic moments pointed in out-of-plane direction which is usually seen for materials with anisotropy with an easy-axis of magnetization in absence of strong in-plane fields. These images can be turned into binary images, and a vast range of similarity metrics can be suitable for these cases. For example, Normalized Cross-Correlation (NCC) or Peak signal-to-noise ratio (PSNR) can give similar phase diagrams as SSIM. Each of the metrics has their limitations. PSNR should be additionally normalized as it can give similarity values $|\xi| > 1$.

NCC metric measures similarity using the mean-normalized cross-correlation between images I_1 and I_2 and is defined as

$$\mathrm{NCC} = \frac{\sum(I_1(i,j) - \bar{I}_1)(I_2(i,j) - \bar{I}_2)}{\sqrt{\sum(I_1(i,j) - \bar{I}_1)^2 \sum(I_2(i,j) - \bar{I}_2)^2}},$$

Where $\bar{I}_1$ and $\bar{I}_2$ are the mean intensities of the images.

PSNR metric is defined as

$$\text{PSNR} = 20 \cdot \log_{10}\left(\frac{\text{MAX}}{\sqrt{\text{MSE}}}\right), \quad \text{MSE} = \frac{1}{mn}\sum_{i=1}^{m}\sum_{j=1}^{n}(I_1(i,j) - I_2(i,j))^2$$

MAX is the maximum possible pixel value (255 for 8-bit images, 1 for normalized images). MSE is the Mean Squared Error between the two images and calculates the average squared pixel-wise difference between two images I_1 and I_2 of size m×n.

For binary images, NCC metric better distinguishes inverted images, which is the case of the same patterns which are completely antiferromagnetically coupled. SSIM does not work well in the cases of completely inverted images, while showing $\xi < 0$ it might still not be -1, as in the case of NCC. However, SSIM works better for gray-scale experimental images than other metrics, as it takes into account structural information. PSNR metric works worth than other metrics, while still showing similar regions in the correlation phase diagram.

References

[S1] Liechtenstein, A. I.; Katsnelson, M. I.; Antropov, V. P.; Gubanov, V. A. Local spin density functional approach to the theory of exchange interactions in ferromagnetic metals and alloys. *Journal of Magnetism and Magnetic Materials* **1987**, *67*, 65–74.

[S2] Szilva, A.; Kvashnin, Y.; Stepanov, E. A.; Nordström, L.; Eriksson, O.; Lichtenstein, A. I.; Katsnelson, M. I. Quantitative theory of magnetic interactions in solids. *Reviews of Modern Physics* **2023**, *95*, 035004.

[S3] Wills, J. M.; Cooper, B. R. Synthesis of band and model Hamiltonian theory for hybridizing cerium systems. *Physical Review B* **1987**, *36*, 3809–3823, Publisher: American Physical Society.

[S4] Wills, J. M.; Alouani, M.; Andersson, P.; Delin, A.; Eriksson, O.; Grechnyev, O. Full-Potential Electronic Structure Method: energy and force calculations with density functional and dynamical mean field theory. *Springer Science & Business Media* **2010**, *167*.

[S5] Grebenchuk, S.; Grzeszczyk, M.; Chen, Z.; Novoselov, K. S.; Koperski, M. Effects of bubble-induced strain on the magnetic properties of van der Waals ferromagnet CrBr3. *Journal of Physics: Materials* **2024**, *7*, 035009.

[S6] Grzeszczyk, M.; Acharya, S.; Pashov, D.; Chen, Z.; Vaklinova, K.; van Schilfgaarde, M.; Watanabe, K.; Taniguchi, T.; Novoselov, K. S.; Katsnelson, M. I.; Koperski, M. Strongly Correlated Exciton-Magnetization System for Optical Spin Pumping in CrBr3 and CrI3. *Advanced Materials* **2023**, *35*, 2209513.

[S7] Wu, J.; Yao, Y.; Lin, M.-L.; Rösner, M.; Du, Z.; Watanabe, K.; Taniguchi, T.; Tan, P.-H.; Haas, S.; Wang, H. Spin–phonon coupling in ferromagnetic monolayer chromium tribromide. *Advanced Materials* **2022**, *34*, 2108506.

[S8] Yang, J.; Wang, A.; Zhang, S.; Liu, J.; Zhong, Z.; Chen, L. Coexistence of piezoelectricity and magnetism in two-dimensional vanadium dichalcogenides. *Physical Chemistry Chemical Physics* **2019**, *21*, 132–136.
